# Common and specific impairments in attention functioning in girls with chromosome 22q11.2 deletion, fragile X or Turner syndromes

**DOI:** 10.1186/1866-1955-6-5

**Published:** 2014-03-14

**Authors:** Andrea I Quintero, Elliott A Beaton, Danielle J Harvey, Judith L Ross, Tony J Simon

**Affiliations:** 1 MIND Institute and Department of Psychiatry and Behavioral Sciences, University of California, Davis, 2825 50th Street, Sacramento, CA 95817, USA; 2 Department of Psychology, University of New Orleans, 2000 Lakeshore Drive, New Orleans, LA 70148, USA; 3 Department of Public Health, Division of Biostatistics, University of California, Davis, One Shields Avenue, Davis, CA 95616, USA; 4 Department of Pediatrics, Thomas Jefferson University, 1025 Walnut Street, Philadelphia, PA 19107, USA

**Keywords:** Attention networks test, Visuospatial cognition, Cognitive development, Developmental disorder, Chromosome 22q11.2 deletion syndrome, Velocardiofacial syndrome, DiGeorge syndrome, Fragile X syndrome, Turner syndrome

## Abstract

**Background:**

Chromosome 22q11.2 deletion syndrome (22q11.2DS), fragile X syndrome (FXS), and Turner syndrome (TS) are complex and variable developmental syndromes caused by different genetic abnormalities; yet, they share similar cognitive impairments in the domains of numbers, space, and time. The atypical development of foundational neural networks that underpin the attentional system is thought to result in further impairments in higher-order cognitive functions. The current study investigates whether children with similar higher-order cognitive impairments but different genetic disorders also show similar impairments in alerting, orienting, and executive control of attention.

**Methods:**

Girls with 22q11.2DS, FXS, or TS and typically developing (TD) girls, aged 7 to 15 years, completed an attention network test, a flanker task with alerting and orienting cues. Exploration of reaction times and accuracy allowed us to test for potential commonalities in attentional functioning in alerting, orienting, and executive control. Linear regression models were used to test whether the predictors of group and chronological age were able to predict differences in attention indices.

**Results:**

Girls with 22q11.2DS, FXS, or TS demonstrated unimpaired function of the alerting system and impaired function of the executive control system. Diagnosis-specific impairments were found such that girls with FXS made more errors and had a reduced orienting index, while girls with 22q11.2DS showed specific age-related deficits in the executive control system.

**Conclusions:**

These results suggest that the control but not the implementation of attention is selectively impaired in girls with 22q11.2DS, TS or FXS. Additionally, the age effect on executive control in girls with 22q11.2DS implies a possible altered developmental trajectory.

## Background

Attention impairments are common to a variety of neurodevelopmental disorders (NDDs), yet their cognitive profiles of specific strengths and weaknesses within the attentional domain remains unclear. Behavioral, anatomical, and neuroimaging studies support the notion that the output of visual attention networks underpins a broad range of complex cognitive skills including representations of space [1], number [2], and social cognition [3]. These higher-order cognitive abilities are dependent upon the foundational development of interacting sensory and perceptual systems that constitute lower-order cognitive systems, including attention. Impairments in lower-order cognitive processing will reverberate through development, affecting the higher-order cognitive systems. These negative consequences are incremental and interactive, as cognitive and emotional systems develop [4]. Studying the development of these processes in children with genetic developmental disorders, whose impairments are related to continuous genetic influence, affords valuable insights into the typical development of the cognitive subsystems that underpin attention, and can provide clarity as to the specific nature of cognitive impairments in the respective NDDs [5].

Chromosome 22q11.2 deletion syndrome (22q11.2DS), fragile X syndrome (FXS), and Turner syndrome (TS) are complex and distinct NDDs that arise from different genetic abnormalities. Physical, intellectual, and cognitive impairments vary both within and amongst these disorders; yet these and other NDDs, such as Williams syndrome (WS), appear to share common cognitive impairments in visuospatial and numerical thinking [6-8]. Cross-syndrome comparisons provide insight into the neurobiological nature of cognitive skills by linking atypically developing behaviors to genetically modulated cellular and anatomical changes. To date, direct behavioral cross-syndrome comparisons have been limited to a single three-way combination of FXS, WS, and Down’s syndrome [9] and a small number of two-way combinations [10-16].

### Etiology of 22q11.2DS, FXS, and TS

A hemizygous deletion on the 22nd chromosome results in 22q11.2DS and occurs in about 1:4,000 live births [17-19]. Standardized neuropsychological testing has established the broad cognitive phenotype of children with 22q11.2DS [20] with mean full-scale intelligence quotient (FSIQ) typically ranging from 70 to 85 [21] with a general strength in verbal relative to nonverbal domains [22] in most individuals. The specificity of these impairments is indicated by group differences on nonverbal tasks of: spatial attention [23,24], executive function [25,26], visuomotor skills [27], visuospatial skills [28], and numerical [23], and arithmetical thinking [29].

FXS results from an expansion of a CGG trinucleotide repeat sequence on the X chromosome. The estimated prevalence is approximately 1:3,600 of male and 1:4,000 of female live births [30]. Due to the process of X inactivation in women, males with FXS have a relatively more severe and less variable presentation of impairments. Female children with FXS show a broad distribution of intellectual function, with approximately 50% displaying borderline to normal FSIQ [31]. Specific impairments have been reported in tasks of: inhibitory aspects of executive function [12,32], spatial relations [33], visuospatial skills [13,34], and arithmetic and numerical processing [35,36].

TS occurs in an estimated 1:2,000 to 1:5,000 live female births [37] due to the partial or complete loss of one X chromosome. FSIQ and verbal comprehension are within the normal range, while perceptual reasoning is lower than in typically developing (TD) girls [38,39]. Additionally, girls with TS demonstrate impairments in several cognitive tests of visuospatial and spatial memory [13], numerical skills [15], executive functions [39], and attention [40].

### Visual attention and the attention network

Posner and colleagues developed the Attention Network Test (ANT) to generate data supporting their proposal that the cognitive processes that constitute attention can be divided into three major subsystems: alerting, orienting, and executive control; each has an associated neuroanatomical network [41-44] and possible molecular basis [45]. Alerting is defined as the state of being sensitive to and maintaining focus on a particular task, item, or location over a period of time. Orienting is the ability to select a characteristic of an item, such as location, over other characteristics, and then shift attention to that aspect. Lastly, executive control selects among competing inputs based on external or internal rules. There are few tests of attention that demonstrate reliability in children, and even fewer that simultaneously test multiple components of attention; see [46]. Nonetheless, previous use of the ANT task in TD children showed the development of these subsystems through late childhood [44,47].

Atypical development in any one of these components of attention will manifest in a general impairment in attention and, perhaps, the higher-order cognitive processes that are built upon attention [8]. Childhood and adolescence, in atypical and typical development, are pivotal times during which cognitive functions mature and improve. The potential for variability in perceptual and cognitive developmental end points is derived from the high degree of neuroplasticity within infancy and early childhood combined with atypical development [5,48], which collectively affect an individual’s perception of and interaction with the world [49,50]. Thus, impairment in visual attention early in development may produce disruptions in visuospatial and numerical processing [6,51]. In turn, impairments in numerical skills, which depend on visuospatial and numerical processing, may arise from impairments in one or a combination of the attention subsystems described by Posner. In other words, different NDDs may exhibit the same cognitive impairment (i.e., in numerical skills) but due to different underlying mechanisms (i.e., have different impaired attention subsystems).

### The present study

Here we examine components of attentional processes in girls with three genetic disorders. The study excludes males because TS is a genetic syndrome that only occurs in females. Second, collapsing across the sexes for FXS is not appropriate, given that behavioral differences are particularly large between the sexes in this population, due to the genetics of the disorder [52,53]. Evidence indicates that between-sex behavioral differences also exist, albeit to a lesser extent in children with 22q11.2DS [54,55]. Finally, sex differences in typical behavior and brain development are well established [56].

The purpose of this study is to identify and describe diagnosis-specific characteristics of the functioning of attention systems in the development of attention subsystems in children with 22q11.2DS, FXS, and TS. It is the first to use a single cognitive test to compare these NDDs. We used the ANT, which is designed to tap into multiple components of attention. We then used linear modeling to examine effects of diagnosis and chronological age on performance across the NDDs.

Our first aim was to determine whether observed attentional challenges arise from general intellectual impairment or from the dysfunction of specific neurocognitive systems. If intellectual impairment best explained the difficulties, then ANT performance should mirror IQ level. More specifically, girls with TS should not perform significantly differently from TD girls, and girls with 22q11.2DS and girls with FXS should both be impaired to a similar degree. If impairments stem from specific cognitive functions unrelated to general intellectual ability, then girls with TS, who typically have an average FSIQ, may differ from TD girls. Alternatively, specific impairments in attention may be due to a common core impairment between these NDDs. If so, one would expect girls with a NDD to perform similarly to each other, but poorer than TD girls. If groups of girls with a NDD perform differently from each other and also poorer than the TD group, this would suggest disorder-specific impairments in the attention network. We hypothesize that for some measures, different NDDs will perform like each other and unlike controls, but that in other measures, different NDDs will exhibit distinct behavioral profiles.

Our final aim was to determine whether age atypically influenced the functioning of attention subsystems, between the ages of 7 and 15, for each NDD. Typical performance on the ANT improves through childhood, but should be stable within the 7 to 10 age range [47]. If the attentional indices develop along a typical time frame, we predict that the performance of girls with NDD will be stable across this age range, perhaps at some lower level of performance.

## Methods

### Participants

A total of 188 children between the ages of 7 and 15 years were recruited as a part of a larger ongoing study. Children with 22q11.2DS or FXS or were TD participated at the MIND Institute at the University of California, Davis. Girls with TS participated at Thomas Jefferson University in Philadelphia. The study was approved by the Institutional Review Boards of Thomas Jefferson University and the University of California, Davis. For all participants, parental consent and child assent were obtained. A subset of the girls in the current study were included in analyses in a prior study using the ANT, including both boys and girls with 22q11.2DS or TD (those data were reported in [57]). Here we present new analyses comparing all female children who are TD or who have a confirmed diagnosis of 22q11.2DS, FXS, or TS with a monosomic 45,X karyotype.

Of the 129 female participants, 42 were TD, 32 had 22q11.2DS, 24 had FXS and 31 had TS. All subsequent statistics are from this female group. Demographic characteristics are reported in Table 1. The mean age in the TD group was 10 years, 3 months (SD =2 years, 3 months), 22q11.2DS group 10 years, 5 months (SD = 2 years), FXS group 11 years, 2 months (SD = 2 years, 3 months), and TS group 10 years, 8 months (SD = 2 years, 4 months). There was no significant difference in age between the four groups (*F*(3,125)=0.89, *P*=0.45). WASI [58], WISC III [59] or WISC IV [60] intelligence quotient (IQ) data were available for 34 TD, 30 22q11.2DS, 21 FXS and 25 TS participants. Characteristics of these measures, including those for processing speed (PS), perceptual reasoning (PRI) and verbal comprehension (VCI), are reported in Table 1.

**Table 1:** Demographic and performance data for subject cohorts

	**TD**		**22q11.2DS**		**FXS**		**TS**	
	**Mean (SD)**	**Range**	**Mean (SD)**	**Range**	**Mean (SD)**	**Range**	**Mean (SD)**	**Range**
Age (years)	10.2 (2.27)		10.4 (1.97)		11.1 (2.27)		10.7 (2.31)	
Error rate^a^	0.03 (0.03)		0.08 (0.12)		0.14 (0.17)		0.07 (0.11)	
SRT	382.3 (85.05)		367.4 (78.37)		428.5 (122.99)		398.5 (90.78)	
FSIQ	112.3 (10.71)	92–135	75.4 (13.74)	52–103	79.5 (20.38)	44–114	96.6 (11.05)	73–118
PS	103.7 (12.77)	80–128	80.6 (13.57)	56–106	78.2 (18.24)	53–115	88.3 (11.92)	62–109
PRI	111.4 (14.38)	87–140	81.0 (13.90)	59–108	88.2 (16.04)	59–120	102.7 (9.53)	85–132
VCI	112.6 (11.03)	92–133	76.5 (14.97)	55–108	80.6 (19.54)	45–115	97.5 (13.77)	73–133

^a^The error rate is the percentage of incorrect trials.

FSIQ, full-scale intelligence quotient; PRI, perceptual reasoning index; PS, processing speed; SD, standard deviation; SRT, simple motor reaction time; VCI, verbal comprehension index.

### Task

**Figure 1:**
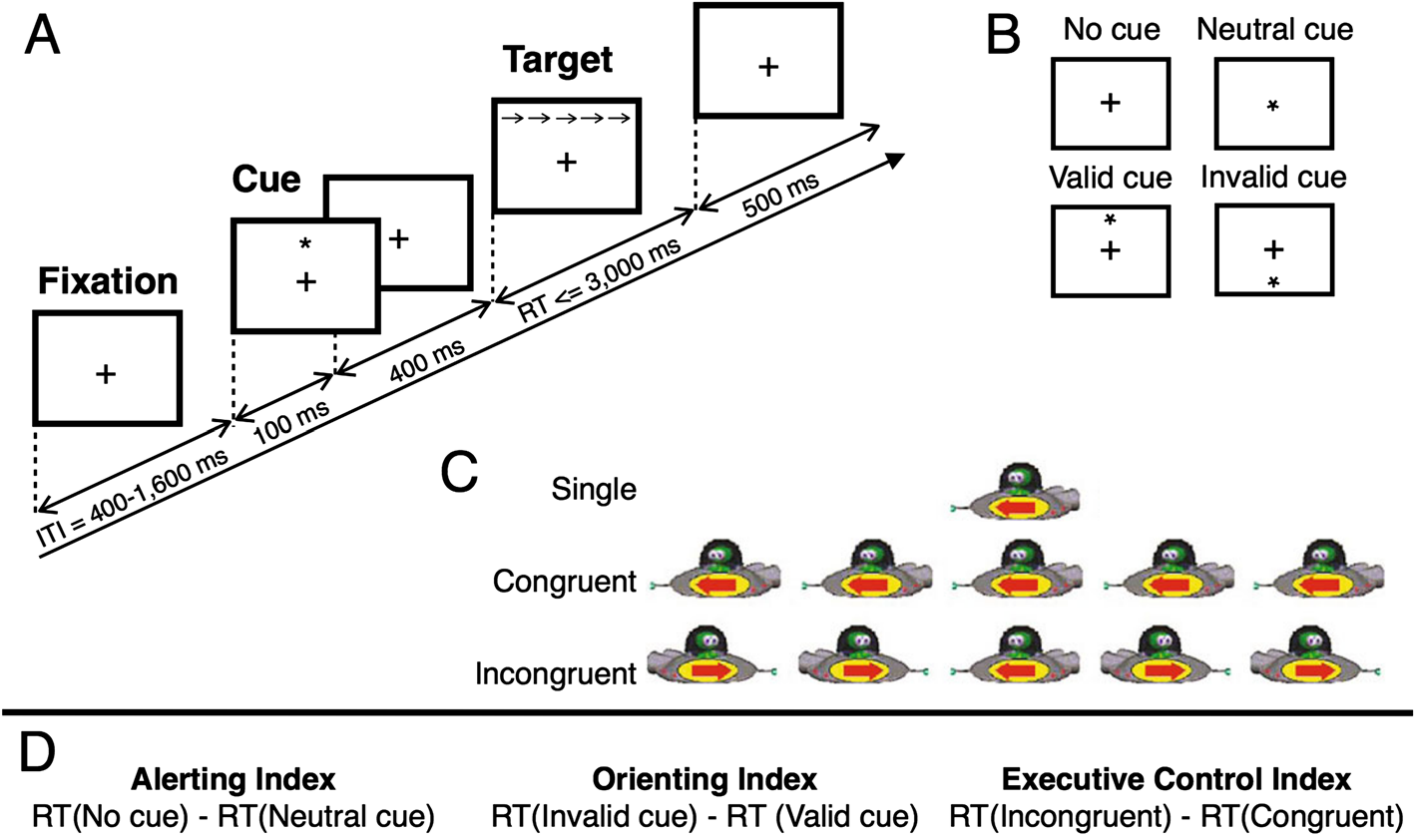
**Outline of experimental task.(A)** Each trial in this children’s version of the attention networks test is made up of the following: an intertrial interval jittered between 400 to 1,600 ms (pseudorandomly distributed at 200 ms intervals), followed by the presentation of a cue stimulus, then after a 400 ms fixation period, the target alien spaceship appears and remains on screen till the child responds or 3,000 ms has passed. **(B)** One of four cue types were presented in each trial. **(C)** The target alien spaceship was centrally presented and could be flanked by other alien spaceships. **(D)** Attentional indices are calculated from the difference score between pairs of conditions. RT, response time.

We used an adaptation of the original children’s ANT [47,57]. The design is illustrated in Figure 1. We used four cue conditions: a centrally located neutral cue, a spatially valid cue, a spatially invalid cue, and no cue. Of the total number of trials, 25% of cues were neutral trials, 37.5% were valid trials, 12.5% were invalid trials, and 25% were no cue trials. The three flanker conditions were presented in equal proportions. The target conditions were: congruent flanking arrows (in the same direction as the target), incongruent flanking arrows (in the opposite direction to the target), and no flankers. Children were asked to respond to the central target arrow by pressing the button that corresponded to the direction the arrow pointed. Primary outcome measures were response time and error rate.

### Data analysis

Data processing and statistical analyses were conducted using R, version 2.11.1 (R Core Team, 2012). Response times (RTs) less than 150 ms were defined as anticipatory responses, and these trials were removed from the analysis. For girls with 22q11.2DS on average 1.5 trials were removed, girls with TS had 2.0 trials removed, girls with FXS had 3.7 trials removed, while TD girls only had 0.1 trials removed. Of the remaining trials, the median RT and percentage of incorrect trials were calculated and used for further analysis. The mean percentage of incorrect trials for each group is listed in Table 1. No participants performed below chance level.

Within each condition, we calculated an adjusted RT by using the formula RT/(1− error rate) to reflect both speed and accuracy for each child. The use of an adjusted RT was done, as before [26], to assess the full performance range of children with NDDs who are known to produce a higher error rate. Using this adjustment, error-free RT remains unchanged at 100% accuracy, and increases in proportion to the number of errors. Such a RT adjustment has been previously used to account for speed accuracy trade-off and reflects the efficiency of a system to perform its calculation successfully [61-63]. Therefore, the main dependent variables for each experimental condition were the median unadjusted RT and median adjusted RT. A participant was defined as an outlier if their median adjusted RT was greater than 2.5 times the interquartile range in a number of conditions. One girl with 22q11.2DS qualified as an outlier for three of the six conditions and on this basis was removed from all further analysis. To assess the contribution of motor reaction time, the average simple motor reaction time (SRT) was measured as part of the larger study in a separate task. Eleven girls did not complete the SRT task: four girls with 22q11.2DS, six girls with TS and one TD girl.

For each individual, we calculated an index of the efficiency of the subsystem’s functioning [42] (Figure 1D). In this study, we will refer to the efficiency of performance (i.e. response time/accuracy within a condition) as attentional efficiency and efficiency of the attentional subsystems (i.e. index score) as network efficiency. The alerting index was calculated by subtracting the median RT of all trials with a neutral cue from the median RT of all trials with no cue. The orienting index was calculated by subtracting the median RT of validly cued trials from the median RT of invalidly cued trials. The executive index was calculated by subtracting the median RT for all congruent trials from the median RT of all incongruent trials. This was done for each individual’s unadjusted RT and adjusted RT.

### Statistical analysis

One-way analyses of variance (ANOVA) were used to test for diagnostic group differences in age, IQ measures and SRT. *Post hoc* comparisons using Tukey’s honestly significant difference (HSD) method were carried out to test mean group differences when the global *F*-test was significant. To test whether the conditions within an index were different from each other, paired *t*-tests within a diagnostic group were used with an alpha level of 0.05 divided by the number of diagnostic groups to account for multiple comparisons within an index.

The effect of diagnosis or age on each attentional index was modeled by linear regression. Due to non-normal distributions of residuals and unequal variances, these outcomes were transformed using the natural logarithm before analysis. Because several participants had indices below zero, 1,500 ms were added to all indices to avoid taking a logarithm of a negative number. To ensure that each intercept was set at the lowest age rather than the absolute minimum age of 0, the age of the youngest participant, 83 months, was subtracted from each individual’s age. All regression models contrasted each NDD group to the TD group. For each attentional index, model building was the same. To address the first aim, simple regression models including only diagnosis were initially fitted to assess unadjusted associations. Joint models were then fitted including both diagnosis and age, in months, or diagnosis and IQ measure. Finally to address the final aim, the interaction between the two factors was added to the joint model.

## Results

### Simple motor reaction time and error rate differences

Group means for SRT and error rates are listed in Table 1. Before testing for group differences, SRTs were natural logarithm transformed due to unequal amounts of within-group variance. There was no significant difference in SRT between the four groups (*F*(3,114)=1.73, *P*=0.16). A Kruskal–Wallis test was conducted to evaluate differences in the error rate between groups across all condition types. The test was significant for an effect of diagnosis (*χ*_2_(3)=10.19, *P*=0.02). A *post hoc* multiple comparison test after a Kruskal–Wallis test with pairwise comparisons indicated that the mean error rate for girls with FXS was significantly higher than for TD girls (*P*=0.01). No other comparisons were statistically different. Analysis of the mean error rates for each index found the same pattern of results.

### IQ measures

Comparisons between each NDD group and the control group showed significant differences on all measures of general cognitive ability. The main effects by group were *F*_FSIQ_(3,106)=45.0 (*P*<0.001), *F*_VCI_(3,106)=30.6 (*P*<0.001), *F*_PRI_(3,106)=38.9 (*P*<0.001) and *F*_PS_(3,101)=19.5 (*P*<0.001). Tukey’s HSD comparison of the four groups indicated that as a group, TD girls had a higher FSIQ, VCI, PRI, and PS than girls with 22q11.2DS (*P*<0.001) and girls with FXS (*P*<0.001). The TD girls also had a higher FSIQ, PRI, and PS compared to girls with TS (*P*<0.001), but did not differ in VCI (*P*=0.08). Among the NDD groups, girls with TS had a higher FSIQ, VCI, and PRI compared to girls with 22q11.2DS (*P*<0.001) and girls with FXS (*P*<0.005). The PS of girls with TS was no different from that of girls with 22q11.2DS (*P*=0.19) or girls with FXS (*P*=0.09).

To test whether having a below average IQ results in a less efficient attentional index, ANOVAs for each unadjusted and adjusted RT attentional index were run. For the alerting index, no significant effect of group (*F*(3,101)=0.71, *P*=0.55), FSIQ score (*F*(1,101)=0.75, *P*=0.38) or group x FSIQ score interaction (*F*(3,101)=1.45, *P*=0.23) was detected. For the orienting index, a significant effect of FSIQ score was indicated (*F*(1,101)=11.65, *P*<0.001), but no significant effect of group (*F*(3,101)=2.22, *P*=0.09) or group x FSIQ score interaction (*F*(3,101)=1.45, *p*=0.23) was detected. For the executive index, a significant effect of group (*F*(3,101)=2.88, *P*=0.04) was indicated, but no significant effect of FSIQ score (*F*(1,101)=0.29, *P*=0.59) or group x FSIQ score interaction (*F*(3,101)=0.61, *P*=0.61) was detected. The same trends were computed using the unadjusted attention indices. Consequently, the initial hypothesis, that ANT performance differences are caused by global impairment to an individual’s mental age and thus are predicted by IQ scores, can be rejected.

### Effect of diagnosis and age on the alerting index

**Figure 2:**
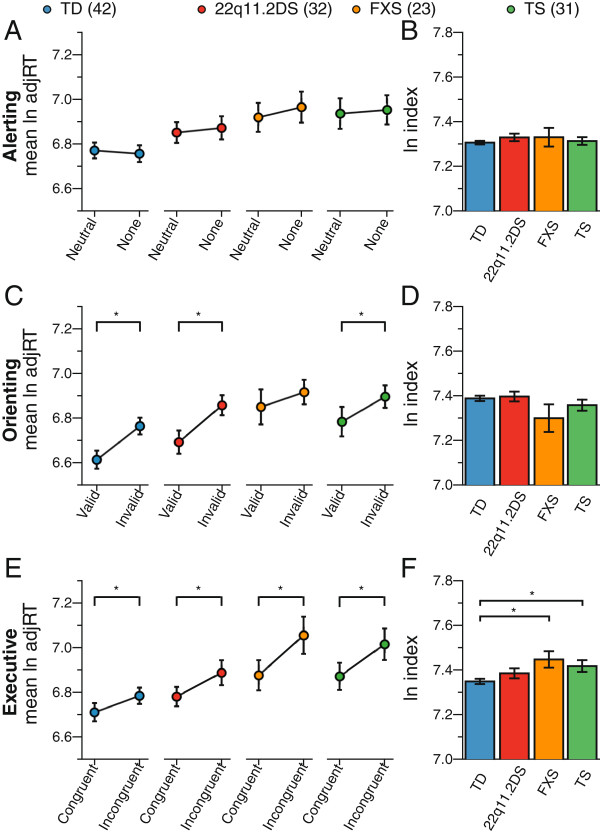
**Analyses of ANT performance for TD girls and girls with 22q11.2DS, FXS, or TS.(A)** Group analyses of response times for the neutral and no cue conditions. Response time to the neutral condition, covaried for age, showed a marginally significant difference in responses for girls with TS compared to TD girls (*P*=0.055). Units are the natural logarithm of the adjusted median response time (ln adjRT). **(B)** Group analysis of alerting index score, covaried for age, showed that girls with a NDD responded similarly to TD girls (*P*=0.61). Index scores are measured as the difference score for each index condition pair. **(C)** Group analyses of response times for the valid and invalid cue conditions. For the adjusted response times to the valid condition, covaried for age, there was a significant difference in responses between girls with TS and TD girls (*P*=0.03). **(D)** Group analysis of orienting index score, covaried for age, showed that girls with a NDD responded similarly to TD girls (*P*=0.35). **(E)** Group analyses of response times for the congruent and incongruent flanker conditions. For the adjusted response times to the congruent condition, covaried for age, there was a significant difference in responses for girls with TS relative to TD girls (*P*=0.02). **(F)** Group analysis of executive control index score, covaried for age, showed that index scores for girls with FXS and girls with TS were significantly larger than for TD girls (*P*=0.07). Error bars represent standard error. 22q11.2DS, chromosome 22q11.2 deletion syndrome; FXS, fragile X syndrome; ln adjRT, natural logarithm of the adjusted median response time; TD, typically developing; TS, Turner syndrome.

For each group, there was no difference in log-transformed adjusted RTs between the neutral cue condition and the no cue condition (Figure 2A): for TD girls *t*=1.22 (*P*=0.23), for girls with 22q11.2DS *t*=−0.91 (*P*=0.37), for girls with FXS *t*=−0.93 (*P*=0.36), and for girls with TS *t*=−0.89 (*P*=0.38). Raw condition means are presented in Table 2. Linear regressions to model behavioral outcome based on group membership revealed no significant differences between groups on the alerting index using either the log-transformed unadjusted RTs (*R*^2^=0.02, *F*(3,124)=0.76, *P*=0.52) or log-transformed adjusted RTs (*R*^2^=0.01, *F*(3,124)=0.37, *P*=0.78). The addition of age to the models did not improve the models’ ability to predict behavioral outcome based on group membership (RT: *R*^2^=0.03, *F*(4,123)=0.83, *P*=0.51; adjusted RT: *R*^2^=0.01, *F*(4,123)=0.29, *P*=0.89) (Table 3 and Figure 2B).

**Table 2:** Average median response times (ms) for subject cohorts

					**Comparison**^**b**^	
**Index**	**Cohort**	***n***	**Mean**^**a**^** (SD)**	**Mean**^**a**^** (SD)**	***t*** **(df)**	***P***
Alerting			Neutral cue	No cue		
	TD	42	862 (200)	861 (213)	1.22 (41)	0.23
	22q11.2DS	31	882 (174)	893 (169)	−0.91 (30)	0.37
	FXS	24	881 (226)	912 (235)	−0.93 (23)	0.36
	TS	31	968 (270)	1000 (292)	−0.89 (30)	0.38
Orienting			Valid cue	Invalid cue		
	TD	42	748 (186)	864 (210)	−7.94 (41)	<0.001
	22q11.2DS	31	761 (164)	913 (201)	−5.67 (30)	<0.001
	FXS	24	825 (248)	880 (196)	−1.28 (23)	0.21
	TS	31	855 (250)	966 (240)	−3.91 (30)	<0.001
Executive			Congruent	Incongruent		
	TD	42	828 (225)	874 (198)	−4.90 (41)	<0.001
	22q11.2DS	31	833 (149)	905 (203)	−3.89 (30)	<0.001
	FXS	24	852 (227)	946 (263)	−4.52 (23)	<0.001
	TS	31	931 (250)	1035 (304)	−4.90 (30)	<0.001

^a^Means of unadjusted individual median response times; ^b^individual adjusted response times were natural logarithm transformed before comparison.

Paired *t*-test, alpha level = 0.0125.

22q11.2DS, chromosome 22q11.2 deletion syndrome; df, degrees of freedom; FXS, fragile X syndrome; SD, standard deviation; TD, typically developing; TS, Turner syndrome.

**Table 3:** Diagnosis and age as predictors of attentional indices

**Linear model estimates of fixed effects on log-transformed (index)**					
**Index**	**Coefficient**	**Estimate ( *β *)**^**a**^	**Standard error**	***P***	**Description**
Alerting					
	Intercept	7.300	0.0234	<0.001	
	22q11.2DS	0.0225	0.0268	0.40	No cost to those with 22q11.2DS
	FXS	0.0234	0.0295	0.43	No cost to those with FXS
	TS	0.0067	0.0271	0.80	No cost to those with TS
	Age (months)	0.00009	0.0004	0.82	No benefit of age
Orienting					
	Intercept	7.372	0.034	<0.001	
	22q11.2DS	0.007	0.0392	0.86	No cost to those with 22q11.2DS
	FXS	0.0234	−0.093	0.03	No cost to those with FXS
	TS	0.0067	−0.033	0.41	No cost to those with TS
	Age (months)	0.00041	0.0006	0.47	No benefit of age
Executive					
	Intercept	7.379	0.034	<0.001	
	22q11.2DS	0.039	0.0307	0.21	No cost to those with 22q11.2DS
	FXS	0.0234	0.0334	0.002	11.3% cost to those with FXS
	TS	0.0067	0.0307	0.02	7.5% cost to those with TS
	Age (months)	−0.00076	0.0004	0.09	No benefit of age

^a^Some parameters were set to 0 as reference for modeling: for the diagnosis category, the typically developing group, and for age, 83 months. 22q11.2DS, chromosome 22q11.2 deletion syndrome; FXS, fragile X syndrome; TS, Turner syndrome.

### Effect of diagnosis and age on the orienting index

As shown in Figure 2C, there was a significant effect of condition type for TD girls and girls with 22q11.2DS or TS, but not for the girls with FXS. Using the log-transformed adjusted RTs, girls responded more efficiently to the valid cue condition than the invalid cue condition (Figure 2C): for TD girls *t*=−7.94 (*P*<0.001), for girls with 22q11.2DS *t*=−5.67 (*P*<0.001), for girls with FXS *t*=−1.28 (*P*=0.21), and for girls with TS *t*=−3.91 (*P*<0.001). Raw condition means are presented in Table 2. Based on linear regression an overall significant difference in the unadjusted orienting index was found between groups (*R*^2^=0.08, *F*(3,124)=3.75, *P*=0.01). The girls with 22q11.2DS had a larger orienting index (*β*=0.02, *t*=1.32, *P*=0.19) compared to the TD girls. However, the girls with TS or FXS had a smaller orienting index (TS: *β*=−0.005, *t*=−0.30, *P*=0.76). The orienting index for girls with FXS was 4% slower compared to TD girls (*β*=−0.04, *t*=−2.32, *P*=0.02). Similar trends were found in the model that used diagnosis and age to predict behavior (*R*^2^=0.09, *F*(4,123)=2.96, *P*=0.02; 22q11.2DS: *β*=0.02, *t*=1.27, *P*=0.21; FXS: *β*=−0.04, *t*=−2.40, *P*=0.02; TS: *β*=−0.006, *t*=−0.36, *P*=0.72). To account for possible speed-accuracy trade-offs, the adjusted orienting RTs were modeled with diagnosis as the sole predictor. Unlike the model of unadjusted RTs, differences in adjusted network efficiency were not predictable (*R*^2^=0.04, *F*(3,124)=1.93, *P*=0.13). The inability of this model to predict group differences, in particular, the significantly smaller orienting index in girls with FXS, may be due to larger variability in the adjusted RTs compared to the unadjusted RTs. The change in variability is probably because there were significantly higher error rates for the valid cue condition for girls with FXS, yet typical error rates for the invalid cue condition. Further, adding age as a predictor to the regression model had no effect on group differences (*R*^2^=0.05, *F*(4,123)=1.57, *P*=0.19) (Table 3 and Figure 2D).

### Effect of diagnosis and age on the executive control index

For each group of girls, there was a significant effect of condition type. Using the log-transformed adjusted RTs, girls responded more efficiently to the congruent flanker condition compared to the incongruent flanker condition (Figure 2E): for TD girls *t*=−4.90 (*P*<0.001), for girls with 22q11.2DS *t*=−3.89 (*P*<0.001), for girls with FXS *t*=−4.52 (*P*<0.001), and for girls with TS *t*=−4.90 (*P*<0.001). Raw condition means are presented in Table 2. Based on linear regression, no overall significant differences in the executive index were found between groups (*R*^2^=0.05, *F*(3,124)=2.09, *P*=0.11). The addition of age to the models did not improve the predictability of group differences using the unadjusted log-transformed RTs (*R*^2^=0.08, *F*(3,123)=1.84, *P*=0.13). Use of the attentional efficiency measure of log-transformed adjusted RTs improved the predictability of the linear model and there was a significant group difference (*R*^2^=0.08, *F*(3,124)=3.41, *P*=0.02). The executive index for girls with 22q11.2DS was larger than for TD girls (*β*=0.04, *t*=1.17, *P*=0.24) and the 10% higher value for girls with FXS and the 7% higher value for girls with TS were significant (FXS: *β*=0.10, *t*=2.95, *P*=0.004; TS: *β*=0.07, *t*=2.23, *P*=0.03). The addition of age to this model generated similar results (*R*^2^=0.10, *F*(4,123)=3.34, *P*=0.01), in which girls with FXS, or TS had significantly worse executive index scores (Table 3 and Figure 2F).

### Effect of age on Attention Network Test indices

Finally, to test for group-specific age effects in each of the ANT indices, we added an interaction term to the attentional index linear regression models. This term allowed us to use the model to explore whether the groups of girls in our cross-sectional sample differed at the age of 83 months, which was the age of our youngest participant. This also provides insight into whether or not age affected the groups differently over time. Linear regression revealed no overall significant difference in the alerting or orienting index between groups (alerting: *R*^2^=0.04, *F*(7,120)=0.67, *P*=0.70; orienting: *R*^2^=0.07, *F*(7,120)=1.68, *P*=0.12). The use of log-transformed adjusted RTs to determine the indices did not improve the predictability of the models (alerting: *R*^2^=0.03, *F*(7,120)=0.46, *P*=0.86; orienting: *R*^2^=0.05, *F*(7,120)=0.88, *P*=0.52).

**Figure 3:**
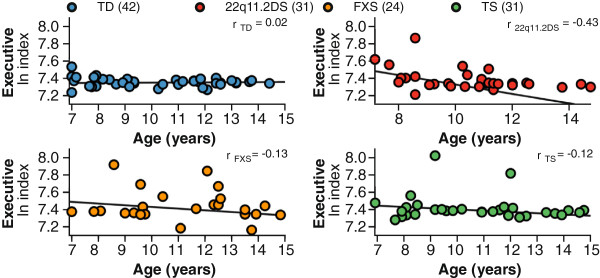
**Analyses of executive control index for TD girls and girls with 22q11.2DS, FXS, or TS.** Individual executive control indices are the difference between the natural logarithms of the adjusted median response times (ln adjRT) for the incongruent and congruent flanker conditions. The executive control index differs in an age-dependent fashion between girls with 22q11.2DS and TD girls (*P*=0.002) for the youngest girls. Cross-sectional analysis revealed a significant annual reduction in the executive control index relative to TD girls (*P*=0.01). There were no significant associations with age for the executive control index for TD girls or girls with FXS, or TS. 22q11.2DS, chromosome 22q11.2 deletion syndrome; FXS, fragile X syndrome; TD, typically developing; TS, Turner syndrome.

Linear regression of the executive index did detect a marginally significant group difference (*R*^2^=0.10, *F*(7,120)=1.98, *P*=0.06). In testing for diagnosis-specific trajectories, we found that the youngest girls with FXS or TS did not have executive indices that significantly differed from the youngest TD girls (FXS: *β*=0.05, *t*=1.41, *P*=0.16; TS: *β*=0.04, *t*=1.48, *P*=0.14); however, the youngest girls with 22q11.2DS had executive indices that were 8% larger than the youngest TD girls (*β*=0.08, *t*=2.64, *P*=0.01). The trajectories for TD girls, girls with FXS and girls with TS were stable and comparable across the age range tested here (TD: *β*=0.0002, *t*=0.46, *P*=0.65; FXS: *β*=−0.0004, *t*=−0.60, *P*=0.55; TS: *β*=−0.0002, *t*=−0.31, *P*=0.76). For girls with 22q11.2DS, a one-year increase in age was associated with a significant 2% reduction in congruency cost compared to TD girls (*β*=−0.0015, *t*=−2.41, *P*=0.02). Adjusted for error rates, the model of executive index was able to account significantly for the variance in performance for the four groups (*R*^2^=0.13, *F*(7,120)=2.43, *P*=0.02). As for the model of unadjusted log-transformed RTs, the youngest girls with 22q11.2DS had executive indices that were 15% larger than the youngest TD girls (*P*=0.025). In this model, for the youngest girls with FXS there was also a significant difference, having a 16% larger executive index compared to the youngest TD girls (*P*=0.031). The trajectories for TD girls, girls with FXS, and girls with TS were stable across the age range tested here (TD: *P*=0.93; FXS: *P*=0.44; TS: *P*=0.52). For girls with 22q11.2DS, a one-year increase in age was marginally significantly associated with a 3% reduction in congruency cost compared to TD girls (*P*=0.06) (Table 4, Figure 3). To determine whether this reduction in network efficiency was driven by an age-related change of attentional efficiency in responding to congruent or incongruent flankers, linear regression models were run that tested for interactions between diagnosis and age. For girls with 22q11.2DS, a one-year increase in age was associated with a 1.5% improvement in attentional efficiency for incongruent flankers (*β*=−0.001), compared to a 8.8% improvement in TD girls (*β*=−0.007), and a 2.5% reduction in attentional efficiency for congruent flankers (*β*=0.002) compared to a 9.3% improvement for TD girls (*β*=−0.007).

**Table 4:** Interaction of diagnosis and age in the executive index

**Linear model estimates of fixed effects on log-transformed (index)**				
**Coefficient**	**Estimate ( *β *)**^**a**^	**Standard error**	*** P***	***Description***
Intercept	7.35	0.0356	<0.001	
22q11.2DS	0.138	0.061	0.025	14.8% cost to those with 22q11.2DS
FXS	0.146	0.0668	0.031	15.7% cost to those with FXS
TS	0.101	0.0571	0.080	No cost to those with TS
Age (months)	0.00007	0.0007	0.929	No benefit of age
22q11.2DS*age	−0.00237	0.0013	0.061	2.8% decrease per year age more than TD
FXS*age	−0.00095	0.0012	0.442	No benefit of age
TS*age	−0.00072	0.0011	0.521	No benefit of age

^a^Some parameters were set to 0 as reference for modeling: for the diagnosis category, the typically developing group, and for age, 83 months.

## Discussion

In the experiment reported in this paper, we found a consistent pattern of results that supports the view that girls with chromosome 22q11.2 deletion syndrome, fragile X syndrome, or 45,X Turner syndrome suffer from both common and diagnosis-specific impairments in visuospatial attention. Despite significant differences in global intellectual functioning between the three groups, girls with 22q11.2DS, FXS, or TS had very similar performance profiles of unimpaired and impaired functioning for the alerting and executive networks. Comparison of the orienting index revealed that only girls with FXS struggle to orient their attention appropriately when presented with valid spatial cues.

### Performance and global intellectual function

Our first aim was to determine whether the observed attentional impairments arose from general intellectual impairment, as designated by IQ, or from specific neurological impairments. We tested the first hypothesis by examining whether between-group differences in behavior and IQ were observed in the same domains. For TD adults, a non-correlation, a significant positive correlation and a significant negative correlation have previously been found between FSIQ and the alerting, orienting and executive indices, respectively [64]. While we were able to replicate the significant positive relation between the orienting index and IQ in the sample as a whole, given the large range of FSIQ in each group, we do not interpret this to mean that based on the FSIQ and orienting index interaction, an individual’s diagnosis group could be predicted.

Intellectual disability is commonly reported in children with 22q11.2DS or FXS, but not in girls with TS. Therefore, if attentional impairment is due to a global intellectual impairment, as represented by reduced IQ, then girls with TS should perform like TD girls and girls with 22q11.2DS or FXS should perform like each other and less well than TD girls. Any other pattern would imply that attention impairment is not linked to global intellectual function. This alternative approach has been studied previously using a different task for children with 22q11.2DS or TS and the results suggested that despite a significant difference in FSIQ between children with 22q11.2DS and girls with TS or TD children, the NDD groups performed more similarly to each other in numerical cognition than to TD children [15]. This supported their interpretation that specific cognitive processing impairments are manifest in these NDDs, and that these differences were not due to differences in IQ. We predicted that our results would replicate this pattern. Here we found that each of the three NDD groups had some form of impaired performance on the ANT. For example, girls with 22q11.2DS, FXS, or TS had significant impairment in the executive control of attention, as had been previously reported for boys and girls with 22q11.2DS [25,57]. This difference in performance is not attributable to the time needed to generate and implement a motor response or to a difference in error rate. Therefore, our results are demonstrably not consistent with the predictions made by a global intelligence hypothesis since significant differences were found between the FSIQ of the groups studied here, but these differences were not replicated in the behavioral differences of an attention task. This suggests that selective impairments in attention reflect either a common core impairment due to the NDD or diagnosis-specific impairments to attentional subsystems.

### Performance and specific neurocognitive function

If girls with a NDD performed differently from each other, and differently from TD girls, this would suggest that there exist diagnosis-specific impairments. For example, the propensity for perseverative behaviors in boys with FXS [12,65] and to a lesser extent in children with 22q11.2DS [66] may contribute to the higher probability that girls with FXS or 22q11.2DS will make incorrect responses. Overall, we predicted both possibilities to be true – in some measures of attention, some NDDs will perform like each other and unlike controls, but that in other measures of attention, different NDDs will exhibit distinct attentional profiles.

The only attentional index in which we found no group differences, no matter which measure we used, was the alerting index. Existing models of attention refer to the ability to direct and maintain focus on an item or location as alerting or sustained attention, a component of which is vigilance [67]. While the definitions may overlap, these concepts are not synonymous with each other. There is a paucity of research comparing cognitive function of attention across NDDs and specifically in girls with 22q11.2DS, FXS, or TS. Evidence suggests that children with 22q11.2DS have impaired sustained attention [66], but unimpaired vigilance [68] and alerting [25,57,69] compared to TD children. A study of boys with FXS using a vigilance task did not find group differences in comparison to boys matched by mental age [9]; however, when the comparison group was divided into those with poor or unimpaired attention, the boys with FXS were slower and less accurate than the boys with better attention [10]. Using a different attention task, adult males with FXS had impaired vigilance [11]. Girls with TS, when compared to children with a learning disability, did not have an impairment in sustained attention [57]; however, when compared to age-matched TD girls, impairments in sustained attention were seen for children [70,71] and adult women with TS [72]. By our approximation, the alerting index is most comparable to other vigilance tasks. Our results are consistent with previous results for children with 22q11.2DS or FXS. To the best of our knowledge, the present study is the first to test girls with TS using a vigilance or alerting task. Our results thus predict that girls with TS will perform comparably to TD girls on a vigilance task.

All three NDD groups had impairments in executive attention when both speed and accuracy were taken into account. Unlike many other tests of executive function that require the selection of an appropriate rule, the ANT primarily requires the selection of the appropriate input to determine the correct response [73], which is made more difficult by the incongruent flankers, as they increase the amount of interference surrounding the informative central arrow. Our findings partially replicate previous studies using the ANT for children with 22q11.DS where the executive but not the alerting or orienting indices were impaired compared to TD control children [25,57,69]. Impairments in other components of executive cognitive function, such as working memory [16] and cognitive flexibility [66], have also been consistently found for 22q11.2DS. Our results therefore concur with this trend. Impairments of executive function in FXS are also well established; however, to our knowledge, this is the first report of the use of a flanker paradigm for this population. For children with FXS, tasks that more directly test cognitive control or resistance to distractors have provided clear evidence of attentional impairment [74], while tasks that require response inhibition or impulse control are commonly impaired for boys with FXS [9,75]. Our results using the ANT suggest that impulse control may also be impaired for girls with FXS. The comparison of error rate also agrees with previous findings of poorer inhibitory control with FXS [12,75]. In the present study, girls with FXS made more errors (10%) than any other groups (less than 1%). Previous studies of girls with TS that tested the relationship between global intellectual functioning and both attentional processing and executive functions found a positive correlation between PRI and executive function [39]. Our analysis did not replicate such a correlation. This could be due to the higher mean IQ of the girls with TS in our study. Another possibility is that the demands of the Attention-Executive function domain subtests of the NEPSY may be more demanding than the ANT. Simultaneous recruitment of both executive function and attention in the context of spatial processing appears to be particularly difficult for girls, adolescents and adults with TS [70,71]. Whether the same is true for attention tasks that require executive function and spatial processing remains to be determined.

While the alerting and executive indices were similar amongst groups, performance on the orienting index was different. Two mechanisms are thought to direct the orientation of attention: voluntary, endogenous shifts of attention and the involuntary, exogenous capture of attention by salient stimuli [76], with the latter maturing earlier than the former [77]. Studies of children with 22q11.2DS have reported impaired endogenous orienting [24] but typical exogenous orienting [25,57,69,78]. While the orienting index is an exogenous cueing, the proportion of valid to invalid cues is not the typical 50:50 ratio. Therefore, our results only resemble previous findings of a typical validity effect, an added benefit to their RTs after the presentation of a valid cue, for 22q11.2DS [25,57,69]. As with girls with 22q11.2DS, the girls with TS in our study responded with a typical validity effect. To the best of our knowledge, no prior studies have specifically measured orienting attention in girls with TS. Interestingly, girls with FXS did not demonstrate any validity effect (i.e., the RTs did not differ between validly and invalidly cued trials). In a follow-up analysis, comparison of adjusted RTs to alerting and orienting cue conditions together revealed no significant facilitation by any cue type for girls with FXS. It is possible that the girls were not using the cue information to assist their responses to the eventual target, as has been seen previously in a mixed gender study [79]. Additionally, in response to an exogenous cueing paradigm, infant boys with FXS performed eye movements as quickly for valid cues as for invalid cues [9]. This indifference to the validity of a cue was also reported in adolescents with FXS, for both exogenous and endogenous orienting [79]. This may also provide insight into the aforementioned higher error rates measured for the FXS group in the current study.

What these three attentional networks have in common is the implementation of selection between competing items or attributes that results in the facilitated processing of what is selected [41,80]. A more recent framework divides attention tasks into those that test the selection between competing inputs or selection between competing rules [73]. Alerting and orienting are clearly examples of processes that place demands on the implementation of selection to use cues effectively, while the executive component, as stated previously, places a higher demand on the implementation of selection, as opposed to the control of selection [81]. This framework aligns itself with proposed neural systems that subserve the attention networks. The alerting and orienting systems are associated with separate regions of the right frontal and parietal lobes [43,82,83] while the executive system is associated with the anterior cingulate and lateral prefrontal cortex [84]. In accordance with our results, volumetric reductions have been noted in children with 22q11.2DS [85] and females with FXS [86]. Though volumetric changes to regions associated with the executive system have not yet been noted in girls with TS, a recent functional connectivity study found reduced functional connectivity with dorsal frontal regions [33]. It can be imagined that these distinct anatomical impairments could result in grossly similar behavioral outcomes within the executive system.

As predicted, behavioral impairments in attention subsystem were confirmed for girls with NDDs. Importantly, not every subsystem was negatively affected and some impairments were restricted to a subset of the NDD groups studied. Given possible anatomical and cellular convergence [6,8,45] between children with 22q11.2DS, FXS, or TS and the distributed nature of behavior, in general, and attention, in particular, it is possible that for each disorder different network ‘nodes’ are perturbed. These perturbations will then interact with the unaffected nodes and disrupt the behavior of higher-order cognitive systems in similar ways, much like a mechanical watch will not keep time correctly if any single cog is misaligned.

### Performance and developmental impairment

Our final aim was to determine whether the development of attention subsystems was delayed in any or each NDD relative to the pattern seen in the TD sample. Studies of typical development consistently demonstrate improvements in the ability to perform increasingly difficult cognitive tests of attention with increasing age [46], generally followed by a period of stabilization [47,87]. For example, there is evidence that the ability to maintain alertness matures around the age of ten [88], while executive control mechanisms continue to develop throughout adolescence and into early adulthood (for a review see [46]). Using the ANT, the indices of the three attentional subsystems in typical development were found to be stable between the ages of six and ten [47]. For children with NDDs, it was not clear whether cognitive impairments stem from late but normal maturation of the requisite neurocognitive system or from a developmental trajectory that stabilizes at a similar rate but poorer level of achievement, despite exposure to relevant stimuli and ensuing practice to build the cognitive skill in question. If the former were the case, then we expected to uncover age-related improvements in performance akin to early stages of development found in younger TD girls. A lack of a significant age-effect in an attentional index implies that stabilization of the attentional index was similarly timed in TD girls and girls with a NDD.

In a cross-sectional analysis and consistent with the findings for TD children, we found no linear age-related changes in the alerting and orienting functions for the sample as a whole or for any of the diagnostic groups. This suggests that for girls with 22q11.2DS, FXS, or TS, the attentional indices for alerting and orienting follow the trajectory of typical development and likely stabilize at ages younger than tested here. For the executive control of attention, in the present study there was no indication of age-related changes for girls with FXS, or TS, like the TD girls. In contrast, the results for girls with 22q11.2DS suggest a potential deviation away from the typical developmental trajectory due to a difference in efficiency between the younger and older girls, evidenced by the smaller differences in RTs between congruent and incongruent flanker conditions in the older participants. Interestingly, for the executive index, a previous study of 22q11.2DS that included both boys and girls, found that age was negatively correlated with RT for incongruent flankers. In this study, where we only included girls in the analysis, this age effect for RT did not have statistical significance. This contrasting finding may highlight gender differences in development for children with 22q11.2DS as we only tested girls in the present study. It is possible that the facilitation by congruent distractors or the interference by incongruent distractors arises from neurocognitive factors relating to gender-specific maturational characteristics, such as increased estrogen in pubertal girls, which affects dopaminergic function [89]. A preliminary analysis comparing the performance of girls grouped by their Tanner stage has suggested this may be the case; however, few girls had reached the later stages at the time of testing. Gender and developmental differences for 22q11.2DS have been understudied, and these results indicate an important direction for future study. Additionally, whether the attentional indices are truly stable in the diagnostic groups during this age range will have to be directly tested through longitudinal studies.

## Conclusions

The goal of this study was to test whether a subset of NDDs shares a common neurobiological impairment that results in complementary behavioral impairments in childhood or adulthood. Using the ANT, the common impairment in attention was differentiated both in comparison to TD age-matched children, but also across the three neurodevelopmental disorders. Atypical brain development is a common factor for children with 22q11.2DS, FXS, or TS, and deficits in higher-order cognitive function, such as mathematical thinking, also appear to be common with these NDDs. However, the origin of these impairments is likely dissimilar just as the nature of the atypical brain development in these NDDs is also dissimilar. We were able to look for generalized and diagnosis-specific strengths and weaknesses. For each disorder, we found that girls did manifest impaired behaviors, but that the exact manifestations were unique for each disorder. We argue that this is still in line with models of shared neurobiological impairment, e.g. [6].

In this study, we investigated whether proposed common foundational impairments in attention for children with 22q11.2DS, TS or FXS underlie shared difficulties with spatial and numerical processing. The efficient functioning of these processes has real implications for navigating a complex world and developing personal agency and autonomy, since people are constantly required to understand spatial (e.g. knowing where to go) and numerical relationships (e.g. being able to cope with simple monetary transactions). It is notable that for task performance, we found more in common between each group of girls with a NDD than not, which suggests that the attentional impairments seen here are more than superficial commonalities. Determining the extent to which these commonalities overlap with neuroanatomical findings [90] is an important avenue of future study.

## Abbreviations

22q11.2DS: chromosome 22q11.2 deletion syndrome; ANOVA: analysis of variance; ANT: Attention Network Test; FSIQ: full-scale intelligence quotient; FXS: fragile X syndrome; HSD: honestly significant difference; IQ: intelligence quotient; NDD: neurodevelopmental disorders; PRI: perceptual reasoning index; PS: processing speed; RT: response time; SRT: simple motor reaction time; TD: typically developing; TS: Turner syndrome; VCI: verbal comprehension index; WS: Williams syndrome.

## Competing interests

The authors declare that they have no competing interests.

## Authors’ contributions

AIQ performed the statistical analyses, interpreted the data, and drafted the manuscript. TJS conceived of and designed the study, acquired the funding, and generated the experimental task. TJS, EAB, and DJH supported the interpretation of the data and revision of the manuscript. JLR recruited the children with Turner syndrome and oversaw the analyses and testing. All authors read and approved the final manuscript.
